# Establishment of a Tissue Culture System for *Quercus palustris*

**DOI:** 10.3390/plants14243870

**Published:** 2025-12-18

**Authors:** Xinyi Wang, Hao Li, Silai Chen, Jinyu Liu, Peng Zhu, Xiaohong Chen, Zhenfeng Xu, Fang He

**Affiliations:** Forest Ecology and Conservation in the Upper Reaches of the Yangtze River Key Laboratory of Sichuan Province, Sichuan Mt. Emei Forest Ecosystem National Observation and Research Station, College of Forestry, Sichuan Agricultural University, Chengdu 611130, China; gulujiujiu@stu.sicau.edu.cn (X.W.);

**Keywords:** *Quercus palustris*, tissue culture, plant growth regulators, micropropagation, in vitro regeneration

## Abstract

*Quercus palustris* possesses significant ecological and ornamental value, yet its clonal propagation remains challenging, hindering germplasm utilization. To address this, an efficient tissue culture propagation system was established. This study systematically evaluated the effects of different plant growth regulator combinations on shoot proliferation, rooting, and callus induction using the Woody Plant Medium (WPM) as the basal culture. The optimal protocol among the tested cytokinin combinations (including 6-benzylaminopurine [6-BA] and kinetin [KT]) for shoot proliferation employed 0.3 mg/L 6-BA and 0.4 mg/L KT, achieving a mean proliferation coefficient of 5.22. For root induction, the most effective treatment consisted of 0.3 mg/L indole-3-butyric acid (IBA) and 0.2 mg/L naphthaleneacetic acid (NAA), yielding a rooting rate of 83.33%. Callus formation was optimally induced by 0.8 mg/L 6-BA combined with 0.3 mg/L NAA, resulting in a high induction rate of 90.63% along with a comparatively low browning incidence of 34.38%. Furthermore, the piperazine derivative fipexide (FPX) exhibited a dual role: promoting callus formation at 10 μmol/L while significantly inhibiting it at concentrations ≥ 20 μmol/L. The established system provides a robust technical foundation for the rapid propagation and germplasm conservation of *Quercus palustris*.

## 1. Introduction

Oaks (genus *Quercus*), also known as oak trees, are naturally distributed across vast regions of Eurasia, Africa, and the Americas, encompassing numerous species of significant ecological and economic importance [1,2,3]. Their wood is prized for its attractive grain and hardness, making it widely used in construction, furniture making, and shipbuilding [4,5]. Furthermore, oak trees play a crucial role in maintaining ecosystem stability, water and soil conservation, and air purification [6,7,8]. *Quercus palustris*, commonly known as the pin oak, is a deciduous tree native to eastern North America. It is notably adapted to wet and even flooded conditions, demonstrating excellent performance in urban greening and ecological restoration projects. Characterized by an upright form, dense foliage, and highly ornamental red autumn coloration, *Q. palustris* has been introduced to several provinces in China for experimental cultivation and landscape use. This species exhibits a considerable stress tolerance, including a degree of resistance to drought, short-term flooding, air pollution, and saline-alkaline soils, indicating a broad habitat adaptation potential. Despite these advantages, the widespread utilization of *Q. palustris* is constrained by immature propagation techniques. Traditional seed propagation is hampered by long cycles, genetic instability, and difficulties in seed storage, which severely limit the development of its germplasm resources and large-scale application [9].

Commonly employed asexual propagation methods for oaks include cutting, grafting, and tissue culture [10]. However, oaks are generally rich in secondary metabolites such as tannins and phenolics, which are prone to oxidative browning, severely compromising explant survival and the rooting process [11]. Conventional cutting propagation often suffers from low survival rates and poor reproducibility, failing to meet the demands of large-scale cultivation. Consequently, in vitro tissue culture is regarded as a crucial pathway for achieving efficient clonal propagation in oaks [12]. This technique not only facilitates the rapid multiplication and conservation of superior genotypes but also provides a platform for subsequent genetic transformation and gene function studies [13]. Successful shoot culture systems have been developed for over 15 oak species, such as *Quercus robur* L. and *Quercus wutaishanica* [14,15]; however, studies on *Q. palustris* propagation remain limited. Significant technical challenges persist, including severe explant browning, low shoot proliferation rates, and unstable root induction. A particular bottleneck is the pronounced exudation of phenolic compounds from shoot tip and nodal explants during culture, leading to medium browning that significantly inhibits organogenesis and plantlet regeneration.

The efficacy of plant tissue culture is influenced by multiple factors, including explant type, medium composition, seasonal growth rhythms, and culture conditions [16,17,18,19]. Regarding hormonal regulation, cytokinins such as 6-benzylaminopurine (6-BA), kinetin (KT), and thidiazuron (TDZ) are commonly employed to promote axillary bud break and shoot proliferation [20,21,22]. In contrast, auxins like indole-3-butyric acid (IBA) and naphthaleneacetic acid (NAA) play a critical role in rhizogenesis [23,24]. Furthermore, as oak explants often harbor endophytic bacteria, the addition of appropriate antibiotics to the culture medium is necessary to suppress contamination; however, the type and concentration of antibiotics require careful optimization to avoid adverse effects on tissue regeneration. In recent years, certain bioactive compounds, such as piperazine derivative, have demonstrated the regulatory potential in plant tissue culture. For instance, the piperazine derivative fipexide (FPX) has been reported to stimulate cell division and callus formation in some species [25]; however, its application efficacy in woody plants, particularly oaks, remains largely unexplored. Investigating the effects of such novel regulators on the micropropagation process of *Q. palustris* is of significant innovative importance for refining its tissue culture protocol.

To address the challenges of low asexual propagation efficiency and an underdeveloped regeneration system in *Q. palustris*, this study employed Woody Plant Medium (WPM) as the basal medium. We systematically optimized the combinations of plant growth regulators (6-BA, KT, NAA, IBA) and investigated the effects of different concentrations of the FPX on callus induction and browning. The aim was to establish an efficient and stable in vitro propagation protocol for *Q. palustris*, encompassing shoot induction with a high proliferation rate, root initiation with a high rooting rate, and callus induction with minimal browning. Among these, the callus induction experiments were designed to establish a platform for biotechnological applications, such as genetic transformation and germplasm conservation.

This research not only provides a reliable technical foundation for the germplasm conservation and commercial micropropagation of *Q. palustris* but also offers valuable theoretical insights and practical references for tissue culture studies in other oak species.

## 2. Materials and Methods

### 2.1. Plant Materials

The plant materials used in this experiment were derived from a single, healthy, disease-free three-year-old *Q. palustris* mother plant obtained from the Huihe Base of Sichuan Agricultural University. Current-year, semi-lignified young stem segments were collected from this plant to serve as explants, ensuring genetic uniformity.

The explants were thoroughly rinsed under running water for 2 h to remove surface impurities. Subsequent surface sterilization was performed under aseptic conditions in a laminar flow cabinet: the explants were first immersed in 75% (*v*/*v*) ethanol for 30 s for initial disinfection, followed by immediate rinsing 2–3 times with sterile distilled water. The explants were then subjected to a sequential sterilization treatment, immersing first in a sodium hypochlorite solution for 15 min and subsequently in a PPM (Plant Preservative Mixture, sourced from Shanghai Maokang Bio-Technology Co., Ltd., Shanghai, China, https://www.maokangbio.com) solution for another 15 min, with gentle agitation throughout. After this treatment, the explants were rinsed thoroughly 4–5 times with sterile distilled water. Finally, surface moisture was blotted dry using sterile filter paper before the explants were inoculated onto the Woody Plant Medium (WPM, LA6880, Beijing Solarbio Science & Technology Co., Ltd., Beijing, China) [26] without plant growth regulators. Sterile shoots obtained from the initial culture were subsequently subcultured for multiplication to expand the population. When the propagated shoots reached a height of ≥2 cm, robust and morphologically uniform apical buds of the stem that was used were selected as experimental materials for subsequent studies.

### 2.2. Medium Preparation and Culture Conditions

The Woody Plant Medium (WPM) was used as the basal medium, supplemented with sucrose (3%, *w*/*v*) as a carbon source and solidified with agar (0.6%, *w*/*v*). The pH was precisely adjusted to 5.80 using 1 mol/L KOH or HCl solutions, followed by autoclaving at 121 °C (approx. 108 kPa) for 20 min.

Plant growth regulators (PGRs)—including KT, 6-BA, IBA, NAA (all in mg/L, all purchased from BIORIGIN, Shanghai, China) and FPX (in μmol/L, Beijing Solarbio Science & Technology Co., Ltd., Beijing, China), and cefotaxime (Cef, in mg/L, obtained from Sigma-Aldrich, St. Louis, MO, USA) were prepared as sterile stock solutions at appropriate concentrations. These stock solutions were filter-sterilized using 0.22 μm microporous membranes and aseptically added to the medium after it had cooled to approximately 60 °C. The medium was mixed thoroughly and then dispensed into sterile plant tissue culture vessels: jars (68 mm in diameter × 94 mm in height) for shoot and root cultures, and Petri dishes (90 mm in diameter) for callus induction. All cultures were maintained in growth chambers under the following conditions: temperature 24 ± 2 °C, relative humidity 70 ± 5%, a 16/8 h (light/dark) photoperiod, and a light intensity of approximately 40 μmol·m^−2^·s^−1^.

### 2.3. Experimental Design

#### 2.3.1. Shoot Proliferation Induced by Different Concentrations of 6-BA and KT

To investigate the effects of different concentration combinations of the cytokinins 6-BA and KT on shoot proliferation, a two-factor completely randomized full factorial design was employed to evaluate the effects of hormone combinations. The experiment comprised nine treatment combinations involving 6-BA (0.3, 0.6, 0.9 mg/L) and KT (0, 0.2, 0.4 mg/L). For each treatment, 15 explants were inoculated, with three replicates per treatment, resulting in a total of 135 samples. Sterile shoots with consistent growth status, bearing 2–3 expanded leaves and an equivalent number of nodes, were selected for inoculation. After 30 days of culture, the following parameters were recorded:

Days to reach 80% lateral shoot initiation rate: In the treatment group, the number of days required for 80% of explants to show lateral shoot emergence.

Proliferation coefficient: This was calculated for each replicate as the total number of lateral shoots (defined as shoots longer than 5 mm) divided by the total number of inoculated explants. Proliferation coefficient = Total number of proliferated shoots/Total number of inoculated explants

#### 2.3.2. Root Induction Induced by Different Concentrations of IBA and NAA

To investigate the effects of different concentration combinations of the auxins IBA and NAA on root induction, a two-factor completely randomized design was employed. The experiment comprised twelve treatment combinations involving IBA (0.3, 0.6, 0.9, 1.2 mg/L) and NAA (0.1, 0.2, 0.3 mg/L). For each treatment, 18 explants were inoculated, with three replicates, resulting in a total of 216 samples. Sterile shoots that were robust and approximately 3 cm in height were selected for inoculation. After 30 days of culture, the rooting rate and the average number of roots, among other indicators, were recorded.

#### 2.3.3. Callus Induction in *Q. palustris* Induced by Different Combinations of 6-BA and NAA and Various Concentrations of FPX

Leaf explants were aseptically excised from one-month-old, rooted *Q. palustris* seedlings grown in a greenhouse or growth chamber. The explants were selected as the 4th to 5th fully expanded leaves from the shoot apex (which were maintained and multiplied on the optimal shoot proliferation medium: WPM supplemented with 0.3 mg/L 6-BA and 0.4 mg/L KT, as established in Section 2.3.1) and subjected to surface scarification. To facilitate contact with the culture medium and induce callus formation, the abaxial (lower) surface of the leaves was gently scarified 3–4 times with a sterile surgical scalpel blade, ensuring cuts were made without severing the leaf. The leaves were inoculated onto the callus induction medium with the adaxial side facing upward and the abaxial side in contact with the medium. Care was taken to ensure consistency in the total number of explants and the total leaf area across different culture vessels. Each culture vessel (Petri dish) was inoculated with a fixed number of five explants. Furthermore, leaf explants of uniform size were selected, and when necessary, were trimmed using a sterile cork borer (8 mm in diameter) to ensure that the total leaf area exposed to the medium was consistent across all replicates. Following inoculation, all cultures were initially maintained in darkness at room temperature for 7 days, and then transferred to a growth room with standard light conditions for an additional 21 days before assessment of callus induction.

To systematically evaluate the effects of different plant growth regulator combinations and FPX concentrations on callus induction from leaf explants, the experimental design comprised two parts. Part I employed a two-factor design, involving twelve combinations (treatments c1–c12) of 6-BA (0.2, 0.4, 0.6, 0.8 mg/L) and NAA (0.1, 0.3, 0.5 mg/L). Part II consisted of a single-factor experiment with FPX, testing six concentration gradients (0, 10, 20, 30, 40, 50 μmol/L; treatments c13–c18), resulting in a total of 18 treatments. For each treatment, five explants were inoculated, with three replicates, yielding a total of 90 explants. Callus induction and growth were monitored periodically. After 21 days of culture, the explant survival rate, callus induction rate, and browning rate were recorded. Browning rate was calculated as the percentage of explants exhibiting significant browning (covering >50% of the explant surface, including partial or total browning) relative to the total surviving explants.

### 2.4. Statistical Analysis

During the tissue culture of *Q. palustris*, experimental data from the shoot proliferation, rooting, and callus induction stages were systematically analyzed. The growth of the plantlets was regularly monitored, and key indicators for each stage were recorded. Statistical evaluation of differences among the treatment groups was performed using one-way analysis of variance (ANOVA) followed by Duncan’s multiple comparison test, to identify the optimal medium formulations and culture conditions.

For shoot proliferation, the following data were collected and analyzed: Lateral shoot initiation dynamics: The time (in days) required for each treatment to reach 80% lateral shoot initiation rate. Lateral shoot count: The total number of lateral shoots per replicate. Proliferation coefficient: Calculated as described in Section 2.3.1. Growth status: Qualitative observations on plantlet vigor, leaf color, and basal callus formation. The lateral shoot initiation rate (percentage of explants initiating shoots at the 80% assessment point) and the proliferation coefficient were compared among treatments to determine the optimal medium for shoot multiplication. For rooting, the rooting time, root number, and thickness were documented. The rooting rate and average root number were compared to identify the best conditions for root induction. For callus induction, the induction process was observed, including the induction rate, callus quantity, growth vigor, and browning rate. The optimal medium for promoting callus formation while suppressing browning was identified accordingly.

## 3. Results

### 3.1. Sterilization Efficiency of Explants

To obtain the sterile shoots of *Q. palustris*, a composite sterilization method using sodium hypochlorite (NaClO) and PPM disinfectant was employed in this study. Different concentration ratios of NaClO and PPM significantly affected the explant contamination rate. When the concentration of NaClO was 10% combined with PPM at 2% (Treatment 9), the contamination rate was the lowest (4.21%). In contrast, when the NaClO concentration was 5% combined with PPM at 0.1% (Treatment 1), the contamination rate was the highest, reaching 45.35%. Overall, the contamination rate showed a decreasing trend with increasing concentrations of both NaClO and PPM (Table 1). By optimizing the sterilization conditions (10% NaClO + 2% PPM), the final contamination rate was controlled below 20%, providing the sufficient sterile materials for subsequent experiments.

### 3.2. Effects of Different Combinations of 6-BA and KT Concentrations on Shoot Proliferation in Q. palustris

**Figure 1:**
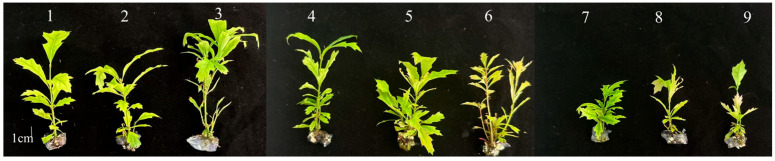
Phenotype of *Q. palustris* shoot proliferation under different combinations of 6-BA and KT after 30 days. Treatment numbers correspond to those in Table 2.

To investigate the regulatory effects of the cytokinins 6-BA and KT on shoot proliferation in *Q. palustris*, this study used robust and morphologically uniform apical buds of the stem (described in Section 2.1) as explants and established nine treatment combinations with different concentration ratios of these hormones. The process of bud germination and proliferation was systematically observed and recorded (Supplementary Figure S1). As shown in Table 2, under treatments with different concentrations of 6-BA and KT, the time required to reach 80% lateral shoot initiation rate ranged from 14 to 18 days, and the proliferation coefficient varied between 1.56 and 5.22, indicating notable differences among the treatments. Among the tested concentrations of 6-BA and KT, treatment a3 (0.3 mg/L 6-BA + 0.4 mg/L KT) exhibited the optimal proliferation effect, achieving an 80% lateral shoot initiation rate in just 13 days, and yielded the highest proliferation coefficient (5.22 ± 0.97). The plants showed the robust growth with compact and nodular callus at the base. In contrast, treatment a9 (0.9 mg/L 6-BA + 0.4 mg/L KT) showed the poorest results, requiring the longest time (18 days) to germinate and producing the lowest proliferation coefficient (1.56 ± 0.73). The plant growth was weak, and callus differentiation was limited (Figure 1 and Figure S2).

**Figure 2:**
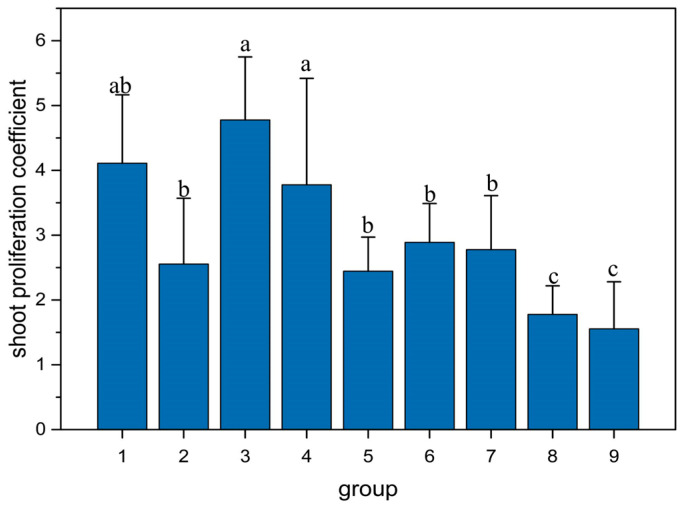
Effects of different combinations of 6-BA and KT concentrations on the proliferation coefficient of *Q. palustris* shoots. Different lowercase letters above the bars indicate the significant differences among treatments at the *p* < 0.05 level according to one-way ANOVA with Duncan’s multiple comparison test. The treatment numbers on the *X*-axis correspond to the following hormone combinations: a1 (0.3 mg/L 6-BA, 0 mg/L KT); a2 (0.3 mg/L 6-BA, 0.2 mg/L KT); a3 (0.3 mg/L 6-BA, 0.4 mg/L KT); a4 (0.6 mg/L 6-BA, 0 mg/L KT); a5 (0.6 mg/L 6-BA, 0.2 mg/L KT); a6 (0.6 mg/L 6-BA, 0.4 mg/L KT); a7 (0.9 mg/L 6-BA, 0 mg/L KT); a8 (0.9 mg/L 6-BA, 0.2 mg/L KT); a9 (0.9 mg/L 6-BA, 0.4 mg/L KT). Data are presented as mean ± standard deviation (*n* = 3).

Furthermore, the multiple comparisons revealed that the proliferation coefficient of treatment a3 was significantly higher than other combinations (Figure 2, Table 2). These results indicate that the appropriate combination of 6-BA and KT can significantly promote shoot proliferation in *Q. palustris*, with the combination of 0.3 mg/L 6-BA and 0.4 mg/L KT being the optimal hormone ratio under the experimental conditions of this study.

### 3.3. Effects of Different Combinations of IBA and NAA Concentrations on Rooting in Q. palustris

**Figure 3:**
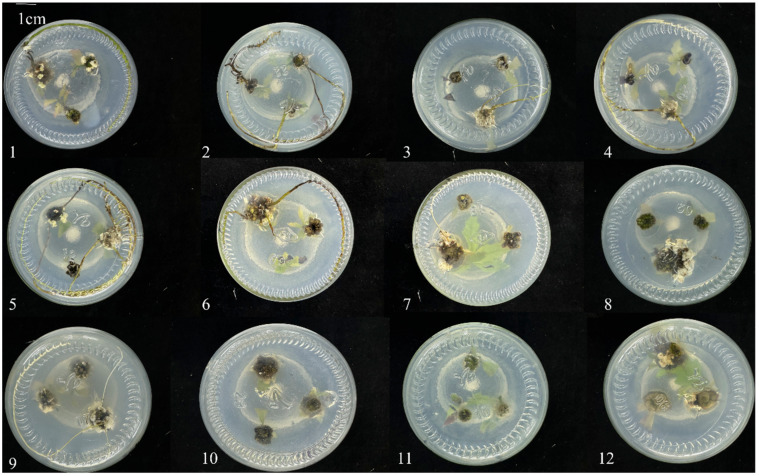
Morphological characteristics of the root system in *Q. palustris* under different IBA and NAA combination treatments after 30 days of culture. Samples 1–12 in the figure correspond to treatments b1–b12 in Table 3, respectively, illustrating the effects of different hormone concentration combinations on root induction number, root thickness, length, and overall development status.

To systematically evaluate the regulatory effects of the auxins IBA and NAA on rooting in *Q. palustris*, this study selected tender shoots from tissue-cultured plantlets with consistent growth status, similar length, and comparable numbers of young leaves as explants (Supplementary Figure S3a,b), establishing a total of 12 treatment combinations of hormone concentrations. The dynamics of root system growth were systematically observed and recorded for plants across all treatments during the culture period. The study demonstrated that different hormone treatments significantly influenced the rooting outcomes. When the IBA concentration was 1.2 mg/L combined with NAA at 0.2 mg/L (Treatment b11), the rooting rate was the lowest, at only 14.29%, with virtually no root development. In contrast, under the combination of 0.3 mg/L IBA and 0.2 mg/L NAA (Treatment b2), the rooting rate reached the highest value of 83.33%, and the root proliferation coefficient was significantly higher than in other treatments (Table 3). Morphological observation indicated that the root systems induced under the optimal treatment (b2, 0.3 mg/L IBA + 0.2 mg/L NAA) were adventitious roots. Careful examination of the explants, as visible, revealed that the majority of roots originated from the callus tissue formed at the base of the microshoots. These roots were thick, exhibited well-developed lateral roots, and were judged to be functional despite their callogenic origin (Figure 3).

**Figure 4:**
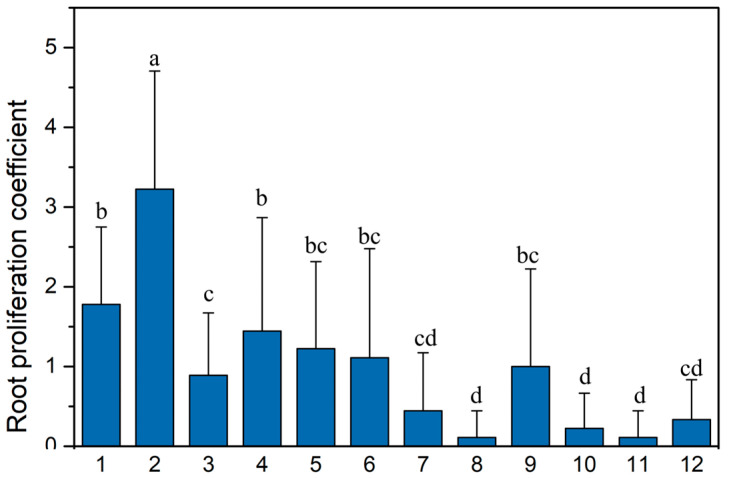
Effects of different combinations of IBA and NAA concentrations on the root proliferation coefficient of *Q. palustris*. Different lowercase letters indicate the significant differences among treatments at *p* < 0.05 level according to one-way ANOVA with Duncan’s multiple comparison test. The treatment numbers (b1-b12) on the *X*-axis correspond to the following hormone concentration combinations (IBA-NAA, in mg/L), as detailed in Table 3: b1 (0.3–0.1); b2 (0.3–0.2); b3 (0.3–0.3); b4 (0.6–0.1); b5 (0.6–0.2); b6 (0.6–0.3); b7 (0.9–0.1); b8 (0.9–0.2); b9 (0.9–0.3); b10 (1.2–0.1); b11 (1.2–0.2); b12 (1.2–0.3). Data are presented as mean ± standard deviation (*n* = 3).

Furthermore, the multiple comparisons indicated that the rooting effect of Treatment b2 was significantly superior to that of other combinations (Figure 4). These results indicate that the combined application of IBA and NAA can effectively induce rooting in *Q. palustris*, with the combination of 0.3 mg/L IBA and 0.2 mg/L NAA being the optimal hormone ratio.

### 3.4. Effects of Different Combinations of 6-BA and NAA Concentrations and FPX on Callus Induction in Q. palustris

To establish an efficient callus induction system for *Q. palustris*, the effects of different concentrations of 6-BA, NAA, and FPX on callus induction from young leaf explants were systematically analyzed.

Based on the statistical analysis of induction rates and browning rates (Table 4), treatment c11 (0.8 mg/L 6-BA + 0.3 mg/L NAA) demonstrated the best callus induction capacity, achieving the highest induction rate of 90.63% while maintaining a relatively low browning rate (34.38%). This indicates that this combination effectively initiates callus formation while suppressing browning. Treatments c10 (0.8 mg/L 6-BA + 0.1 mg/L NAA) and c14 (10 μmol/L FPX) showed the most effective control of browning, with browning rates of 22.73and 21.05%, respectively, indicating the good potential for reducing browning. In contrast, the treatments with high concentrations of NAA (e.g., c3, c9) or FPX (e.g., c18) exhibited severe browning; treatment c18 had a browning rate as high as 88.89%, significantly impairing normal callus growth. Furthermore, treatments c7 and c10 resulted in the highest survival rates of leaf explants, suggesting that an appropriate ratio of cytokinin to auxin (0.8 mg/L 6-BA and 0.3 mg/L NAA) helps maintain explant viability, providing a good foundation for callus formation.

Morphological differences in callus were evident among the treatments (Supplementary Figure S4). Various combinations of 6-BA and NAA, particularly treatments c10 (0.8 mg/L 6-BA + 0.1 mg/L NAA) and c11 (0.8 mg/L 6-BA + 0.3 mg/L NAA), induced compact, granular callus that was predominantly light green in color, with minimal browning, indicating healthy cell status. In contrast, FPX treatments, especially at higher concentrations (e.g., c18, 50 μmol/L FPX), resulted in loose, friable callus that was light yellow to dark brown, exhibiting significant browning and necrosis (Table 4).

These results indicate that the combination of 6-BA and NAA is significantly more effective than FPX treatments for callus induction in *Q. palustris*. The ratio of 0.8 mg/L 6-BA and 0.3 mg/L NAA is optimal, achieving a high induction rate while effectively controlling the degree of browning.

## 4. Discussion

Regarding shoot proliferation, the study found that, among the tested concentrations of cytokinins, the combination of 0.3 mg/L 6-BA and 0.4 mg/L KT yielded the optimal proliferation efficacy for *Q. palustris*. The resulting plants exhibited the robust growth with basal callus formation, demonstrating the favorable morphogenetic potential. This result indicates that the synergistic action of low concentrations of 6-BA and KT effectively promotes bud differentiation and growth. In contrast, a high concentration of 6-BA (0.9 mg/L) exerted an inhibitory effect, leading to a significantly reduced proliferation coefficient (1.56–1.78) and poor plant growth. This finding is consistent with research on *Q. robur*, where a higher proliferation coefficient was achieved on WPM medium supplemented with 0.3 mg/L 6-BA [14]. In our study, the optimal response was achieved not with 6-BA alone, but with a specific combination of 0.3 mg/L 6-BA and 0.4 mg/L KT. The fact that the addition of 0.2 mg/L KT to 0.3 mg/L 6-BA (a2) resulted in a lower proliferation coefficient than 6-BA alone (a1) underscores the complexity of cytokinin interaction and the critical importance of identifying the optimal ratio, rather than simply adding KT. This phenomenon aligns with reports that excessively high cytokinin levels can disrupt apical dominance and induce excessive callus formation [27,28], suggesting that the balance between cytokinins and auxins is crucial for maintaining of normal bud development [29]. The optimal cytokinin balance (0.3 mg/L 6-BA + 0.4 mg/L KT) for shoot proliferation under the experimental conditions of this study in *Q. palustris* exhibits species-specific characteristics when compared to other oak species. For example, in *Q. robur*, lower cytokinin concentrations are sufficient for effective shoot proliferation [14]. This phenomenon aligns with reports in *Q. suber*, where high cytokinin levels can disrupt development [30]. These interspecific variations highlight the importance of tailored hormonal regimens, potentially arising from differences in endogenous hormone levels, receptor sensitivity, or signal transduction pathways among tree species [31,32].

In root induction, the combination of 0.3 mg/L IBA and 0.2 mg/L NAA was most effective, achieving a rooting rate of 83.33% and producing the robust root systems with well-developed lateral roots. This is consistent with findings in *Q. robur* [14]. IBA and NAA, both auxins, differ in their metabolic stability and transport: The NAA is less susceptible to degradation, providing a sustained effect, while IBA is more readily transported within tissues [33]. Their combination likely acts synergistically, with NAA ensuring stable root primordia initiation and IBA facilitating auxin distribution to optimize rhizogenesis [34,35]. High auxin concentrations (e.g., IBA ≥ 0.9 mg/L) significantly inhibited rooting, possibly due to auxin-induced ethylene synthesis or reactive oxygen species accumulation, which can impede root differentiation [36,37]. The addition of NAA in this study significantly enhanced both the rooting rate and root quality, potentially attributable to the differences in stability, transport mechanisms, and metabolic pathways between NAA and IBA [38].

Regarding callus induction, the combination of 0.8 mg/L 6-BA and 0.3 mg/L NAA was the most effective, achieving an induction rate of 90.63%. The induced callus was compact, exhibited a healthy light-green color, and had a relatively low browning rate (34.38%). The FPX at a concentration of 10 μmol/L showed a moderate promotive effect (induction rate 63.16%); however, concentrations exceeding 20 μmol/L resulted in significant inhibition (induction rate ≤ 11.11%) accompanied by high browning rates (up to 88.89%). This indicates that the synergistic effect of 6-BA and NAA is crucial for callus formation, whereas the application of FPX requires strict concentration control. This result is superior to the callus induction efficiency reported by Corredoira et al. for *Q. robur* leaves (approximately 70%), but lower than their induction efficiency for somatic embryos from shoot tips (exceeding 95%) [15], suggesting that explant type, developmental stage, and genotype are key factors influencing callus induction efficiency [39,40]. The FPX slightly promoted callus formation at 10 μmol/L, but higher concentrations (≥20 μmol/L) significantly suppressed callogenesis and exacerbated browning. This concentration-dependent dual effect suggests that piperazine derivative signaling may play complex roles in regulating both cell proliferation and stress responses. Based on its compact and granular morphology, which is a well-documented characteristic of embryogenic callus in many plant species [41,42], the callus induced by the 6-BA and NAA combination is suggested to possess the potential of embryogenic callus. Conversely, the loose and friable morphology of FPX-induced callus is typically associated with non-embryogenic states [19]. This reflects how different regulatory substances influence callus type and quality by affecting the cell cycle, energy metabolism, or secondary metabolite synthesis pathways [43]. Furthermore, browning is a common issue in tissue culture of oaks [30]. The loose, highly susceptible-to-browning callus induced by FPX treatments highlights the differential impact of various regulatory substances on cellular metabolic pathways. The high browning rates observed in several treatments in this study may be related to phenolic oxidation and programmed cell death [44].

While this study successfully established a robust in vitro propagation protocol for *Q. palustris*, encompassing efficient shoot proliferation, root induction, and callus formation, the critical process of acclimatization—the transition of plantlets from in vitro to ex vitro conditions—was not within the scope of this initial phase. The primary focus here was to systematically overcome the major in vitro bottlenecks specific to this genus, such as phenolic browning and low organogenic response, and to define the optimal hormonal requirements for each developmental stage. The high-quality root systems achieved through direct organogenesis (as discussed in Section 3.3), characterized by their robustness and basal callus, provide a strong foundation for successful acclimatization in future applications [45,46]. Future work will prioritize acclimatization trials to validate the practical applicability of this protocol. This represents the essential next step towards the commercial micropropagation and large-scale conservation of *Q. palustris*.

The significance of this optimized propagation system extends beyond overcoming in vitro bottlenecks, offering substantial value for the sustainable management of *Q. palustris*. The capability for efficient clonal propagation addresses a major limitation in the commercial deployment of superior genotypes, enabling the large-scale production of trees with desired ornamental and ecological traits. From a conservation perspective, this protocol serves as a vital tool for the ex situ preservation of genetic diversity, helping to safeguard the species’ adaptive potential against environmental threats [47]. Moreover, the establishment of a reliable regeneration system, particularly the induction of embryogenic callus, lays the groundwork for future genetic improvement strategies, potentially enabling the introduction of novel traits through advanced biotechnologies [41,48]. Consequently, this study provides a comprehensive technical foundation that significantly advances the propagation, conservation, and potential biotechnological enhancement of *Q. palustris*.

## 5. Conclusions

This study established an efficient propagation system for *Q. palustris* through systematic screening of plant growth regulator combinations. The results demonstrated that for the shoot proliferation stage, the optimal combination of the cytokinins 6-BA and KT was 0.3 mg/L 6-BA + 0.4 mg/L KT, achieving a proliferation coefficient of 5.22 ± 0.97. For the rooting stage, the most effective treatment was 0.3 mg/L IBA + 0.2 mg/L NAA, yielding a rooting rate of 83.33%. For callus induction, the most suitable combination was 0.8 mg/L 6-BA + 0.3 mg/L NAA, resulting in an induction rate of 90.63% with a relatively low browning rate (34.38%). Furthermore, FPX at 10 μmol/L exhibited a promotive effect on callus formation, whereas higher concentrations (≥20 μmol/L) significantly inhibited callogenesis and exacerbated browning. This optimized system significantly enhances the efficiency and quality of in vitro propagation of *Q. palustris*, providing a reliable platform for its germplasm conservation and commercial micropropagation. The established protocol, particularly the high-frequency rooting system, establishes a solid foundation for subsequent acclimatization trials, which will be essential for validating the complete clonal propagation cycle and enabling practical application. Moreover, the methodology offers a valuable reference for developing tissue culture protocols for other oak species. Future studies should focus on elucidating the molecular mechanisms underlying hormonal regulation, as well as the interactive effects between environmental factors (e.g., light and temperature) and phytohormones, to further refine the tissue culture system for *Q. palustris*.

## Figures and Tables

**Table 1:** Effects of Different Concentrations of NaClO and PPM on Explant Contamination Rate Note: Contamination rate (%) = (Number of contaminated explants/Total number of inoculated explants) × 100%. A total of 30 explants were inoculated per treatment group.

Treatment Group	NaClO Concentration (%)	PPM Concentration (%)	Contamination Rate (%)
1	5	0.1	45.35
2	7.5	0.1	23.86
3	10	0.1	17.88
4	5	1	15.5
5	7.5	1	12.88
6	10	1	10.67
7	5	2	8.64
8	7.5	2	6.46
9	10	2	4.21

**Table 2:** Effects of Different Concentrations of 6-BA and KT on Lateral Shoot Proliferation in *Q. palustris* Note: Data are presented as mean ± standard deviation (*n* = 3). Proliferation coefficient = Total number of proliferated shoots/Total number of inoculated explants.

Treatment No.	6-BA (mg/L)	KT (mg/L)	Days to Reach 80% Lateral Shoot Initiation Rate
a1	0.3	0	15
a2	0.3	0.2	16
a3	0.3	0.4	13
a4	0.6	0	15
a5	0.6	0.2	16
a6	0.6	0.4	15
a7	0.9	0	14
a8	0.9	0.2	15
a9	0.9	0.4	18

**Table 3:** Effects of Different Concentrations of IBA and NAA on Root Proliferation in *Q. palustris*. Note: Data are presented as mean ± standard deviation (*n* = 3). Root proliferation coefficient = Total number of roots produced/Number of inoculated plantlets.

Treatment No.	IBA (mg/L)	NAA (mg/L)	Total Number of Roots	Rooting Rate (%)
b1	0.3	0.1	16	66.67
b2	0.3	0.2	20	83.33
b3	0.3	0.3	14	66.67
b4	0.6	0.1	12	50.00
b5	0.6	0.2	15	62.50
b6	0.6	0.3	12	57.14
b7	0.9	0.1	10	41.67
b8	0.9	0.2	5	23.81
b9	0.9	0.3	7	33.33
b10	1.2	0.1	6	25.00
b11	1.2	0.2	3	14.29
b12	1.2	0.3	10	41.67

**Table 4:** Comprehensive Effects of Different Treatments on Leaf Survival, Callus Formation, and Browning in *Q. palustris.* Note: Induction rate (%) = (Number of leaves with callus growth/Number of surviving leaves) × 100%; Browning rate (%) = (Number of browned explants/Number of surviving leaves) × 100%. Data are based on statistical results after 21 days of culture under light conditions.

Treatment No.	6-BA (mg/L)	NAA (mg/L)	FPX (μmol/L)	Number of Surviving Leaves	Number of Leaves with Callus Growth	Induction Rate (%)	Browning Rate (%)
c1	0.2	0.1	-	29	3	10.34	86.21
c2	0.2	0.3	-	14	9	64.28	42.86
c3	0.2	0.5	-	13	7	53.85	76.92
c4	0.4	0.1	-	35	20	57.14	42.86
c5	0.4	0.3	-	4	1	25.00	100.00
c6	0.4	0.5	-	15	10	66.67	40.00
c7	0.6	0.1	-	43	29	67.44	27.91
c8	0.6	0.3	-	32	19	59.38	43.75
c9	0.6	0.5	-	55	26	47.27	69.09
c10	0.8	0.1	-	44	32	72.73	22.73
c11	0.8	0.3	-	32	29	90.63	34.38
c12	0.8	0.5	-	49	34	69.38	36.73
c13	-	-	0	13	1	7.69	0.00
c14	-	-	10	38	24	63.16	21.05
c15	-	-	20	39	2	5.13	28.21
c16	-	-	30	35	3	8.57	34.29
c17	-	-	40	24	1	4.17	58.33
c18	-	-	50	9	1	11.11	88.89

## Data Availability

Data will be made available upon request.

## Supplementary Materials

The following supporting information can be downloaded at https://www.mdpi.com/article/10.3390/plants14243870/s1, Supplementary Figure S1. Morphological changes in explants during shoot proliferation under different 6-BA and KT treatments. Supplementary Figure S2. Representative phenotypes of *Q. palustris* shoot proliferation under different hormone treatments after 30 days of culture. Supplementary Figure S3. Explants subjected to IBA and NAA treatments. Supplementary Figure S4. Comparison of callus induction and browning in *Q. palustris* leaf explants under different combinations of plant growth regulators and FPX.
