# Supplementary material for: Establishment of a Tissue Culture System for Quercus palustris
Source: Plants (Basel). 2025 Dec 18;14(24):3870. doi: 10.3390/plants14243870 (PMC12736435; doi:10.3390/plants14243870)
Supplement: Supplementary file 1 [file plants-14-03870-s001.zip › plants-3994122-supplementary.pdf]

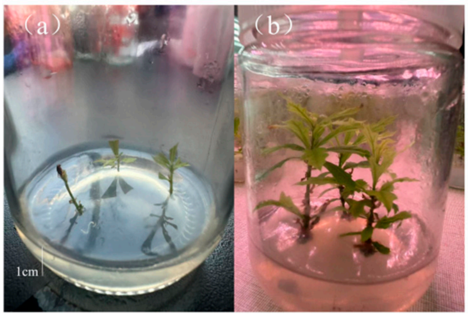

Supplementary Figure S1. Morphological changes of explants during shoot proliferation under different 6-BA and KT treatments. (a) Representative young leaf segments of the uniform apical buds explants at the initiation of culture (0 days). (b) Representative axillary buds and associated young leaves of the explants after 30 days of culture, showing lateral shoot outgrowth. (Scale bar = 1 cm).

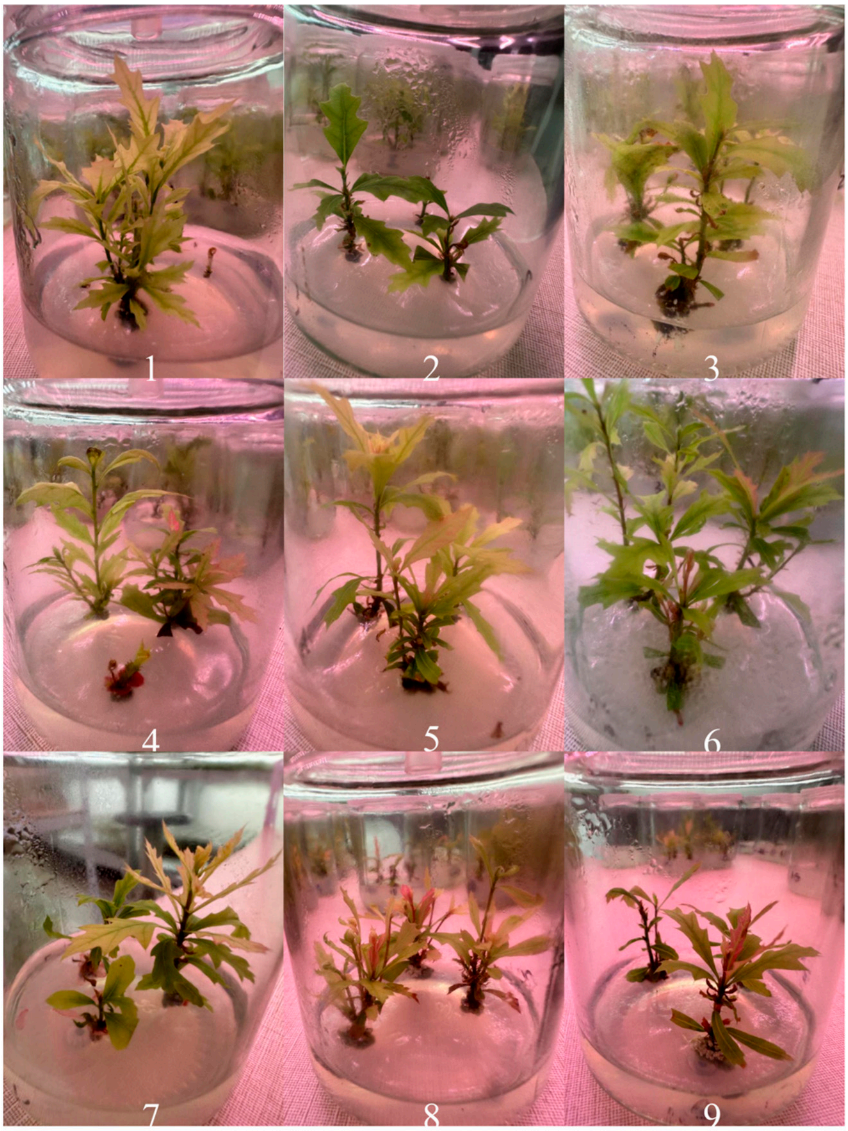

Supplementary Figure S2. Representative phenotypes of *Q. palustris* shoot proliferation under different hormone treatments after 30 days of culture. Numbers 1-9 in the figure correspond to treatments a1-a9 in Table 5, respectively. Differences among treatments regarding the number of lateral shoots, plant vigor, leaf color and morphology, and basal callus formation are compared.

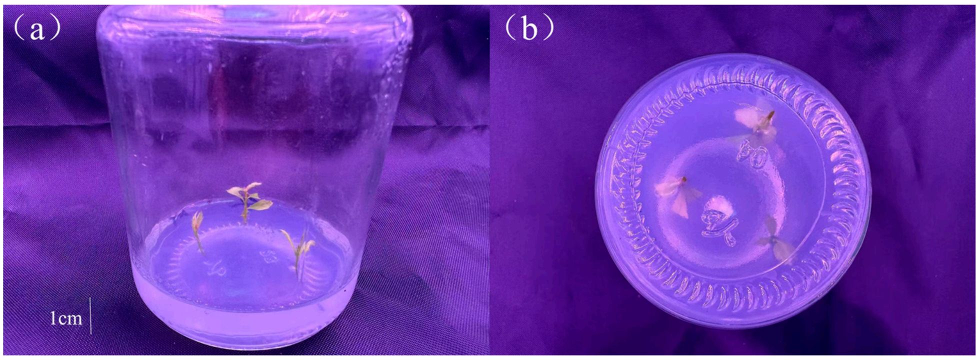

Supplementary Figure S3. Explants subjected to IBA and NAA treatments. The figure shows the morphology of *Q. palustris* explants at the initiation day (0 days) of root induction culture. (a) Young leaf portion used for inoculation; (b) Morphology of the explant base, showing the initial state at inoculation. (Scale bar = 1 cm)

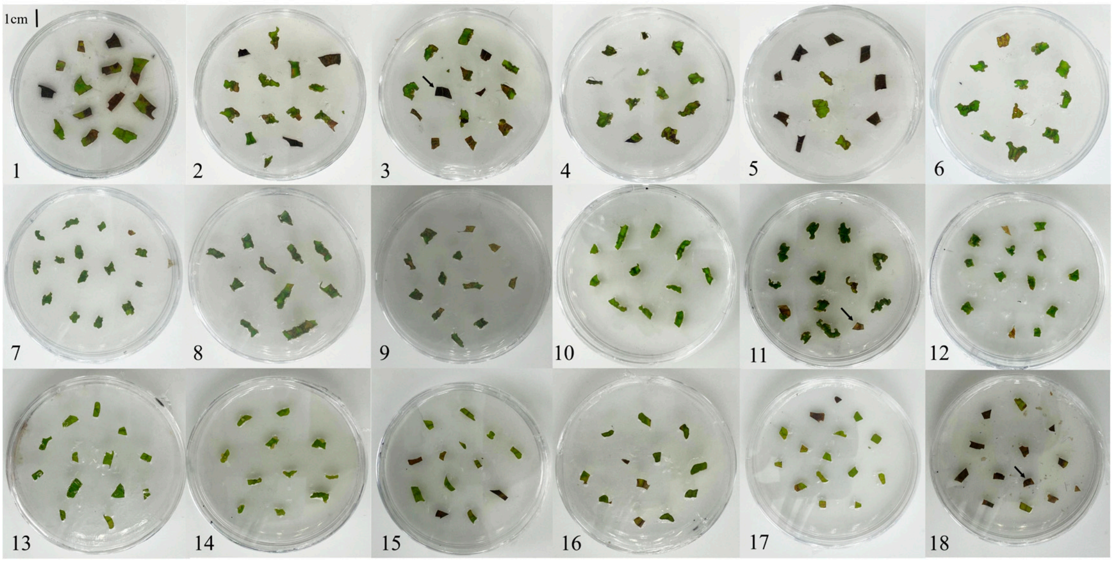

Supplementary Figure S4. Comparison of callus induction and browning in *Q. palustris* leaf explants under different combinations of plant growth regulators and FPX. Samples 1-18 correspond to treatments c1-c18 detailed in Supplementary Table S3. White arrows in the figure highlight key morphological differences: Treatment c11 (0.8 mg/L 6-BA + 0.3 mg/L NAA) induced large, compact, and light green callus (→), indicating healthy growth and high embryogenic potential. In contrast, treatment c18 (50 μmol/L FPX) resulted in severely browned and necrotic tissues with minimal callus formation (→), demonstrating a strong inhibitory effect. Treatment c3 (0.2 mg/L 6-BA + 0.5 mg/L NAA) exhibited intermediate callus growth with noticeable

browning (→), representative of suboptimal conditions. These annotations serve to clearly identify critical phenotypic responses, particularly for print versions where color discrimination may be limited. (Scale bar = 1 cm)
